# Sphingosine-1-phosphate in the regulation of diabetes mellitus: a scientometric study to an in‐depth review

**DOI:** 10.3389/fendo.2024.1377601

**Published:** 2024-12-24

**Authors:** Jieying Li, Yongfu Fan, Wenling Tu, Linyue Wu, Yun Pan, Mingze Zheng, Yiqian Qu, Lingyong Cao

**Affiliations:** School of Basic Medical Sciences, Zhejiang Chinese Medical University, Hangzhou, Zhejiang, China

**Keywords:** sphingosine-1-phosphate, type 2 diabetes mellitus, bibliometric analysis, lipid metabolism, insulin sensitivity, inflammatory responses

## Abstract

Diabetes is a significant global health issue, causing extensive morbidity and mortality, and represents a serious threat to human health. Recently, the bioactive lipid molecule Sphingosine-1-Phosphate has garnered considerable attention in the field of diabetes research. The aim of this study is to comprehensively understand the mechanisms by which Sphingosine-1-Phosphate regulates diabetes. Through comprehensive bibliometric analysis and an in-depth review of relevant studies, we investigated and summarized various mechanisms through which Sphingosine-1-Phosphate acts in prediabetes, type 1 diabetes, type 2 diabetes, and their complications (such as diabetic nephropathy, retinopathy, cardiovascular disease, neuropathy, etc.), including but not limited to regulating lipid metabolism, insulin sensitivity, and inflammatory responses. This scholarly work not only unveils new possibilities for using Sphingosine-1-Phosphate in diabetes treatment but also offers fresh insights and recommendations for future research directions to researchers.

## 1 Introduction

Defined as a chronic metabolic disorder, Diabetes Mellitus (DM) is shaped by various causal factors and shows disturbances in glucose and lipid metabolism. Projections indicate that by 2035, the global prevalence of diabetes will reach 592 million, with over 500 million cases of Type 2 Diabetes Mellitus (T2DM), representing a significant health threat worldwide (1). Contemporary medical research reveals that the pathogenesis of diabetes involves complex interactions including insulin resistance, defects in insulin secretion, viral infections, inflammatory responses, and gastrointestinal effects, significantly influencing its development and progression. Through intricate biological processes, elements such as hyperglycemia, obesity, and abnormal cholesterol and triglyceride levels propel the progression of prediabetes (2, 3). If untreated, DM patients may experience prolonged hyperglycemia, which impairs microcirculation and can result in severe vascular and neuropathic complications, potentially leading to death or disability. Consequently, delaying the onset and progression of DM has become a significant concern for the global medical community.

Sphingosine-1-Phosphate (S1P) results from the phosphorylation of sphingosine, catalyzed by two isoenzymes, SphK1 and SphK2 (4, 5), of Sphingosine kinase. S1P is recognized as a critical regulator in various physiological and pathological processes, including cancer, cardiovascular health, and diabetes (6). S1P induces multiple biological effects, such as cell motility, differentiation, survival, inflammation, immunity, calcium homeostasis, and angiogenesis (7). It is widely recognized that numerous biological effects of S1P are mediated through its interaction with five specific G protein-coupled receptors located on cell surfaces or within cells (8). Moreover, S1P impacts adipocyte differentiation as well as the synthesis and breakdown of fatty acids, altering the balance between lipid storage and energy expenditure (9). Activation of S1P receptors enables S1P to affect lipoprotein lipase activity, directly impacting fatty acid mobilization and storage in adipose tissues. Studies have demonstrated that S1P promotes lipid accumulation in adipocytes, contributing to the development of obesity (10). Additionally, S1P controls glycogenolysis and gluconeogenesis, crucial processes in managing obesity and diabetes (11). Changes in S1P levels in obese and diabetic individuals can result in abnormalities in downstream insulin receptor signaling pathways, such as PI3K/Akt, thus impacting insulin sensitivity and fostering the development of insulin resistance (12). S1P contributes to managing chronic inflammation in obesity by affecting the migration and activation of inflammatory cells, as well as the production of pro- and anti-inflammatory cytokines (13). In conclusion, S1P has emerged as a significant focus in diabetes research.

Bibliometric analysis uses statistical and mathematical techniques to explore relationships and trends among knowledge carriers, clarifying the dynamic attributes of scientific fields and offering valuable references for both foundational and practical research (14, 15). This study employs visualization tools like VoSviewer (16) and CiteSpace (17) to scrutinize and assess existing literature. Furthermore, bibliometric techniques are extensively applied across diverse medical disciplines, such as surgery, oncology (18), neurology (19), and pain medicine (20), to pinpoint research trends and recommend future directions. By statistically analyzing bibliometric data concerning the role of sphingosine-1-phosphate in diabetes regulation, this study synthesizes key research themes and identifies emerging trends, establishing a solid theoretical base for future research on S1P and diabetes.

## 2 Scientometric study

### 2.1 Literature search, screening and download

**Figure 1:**
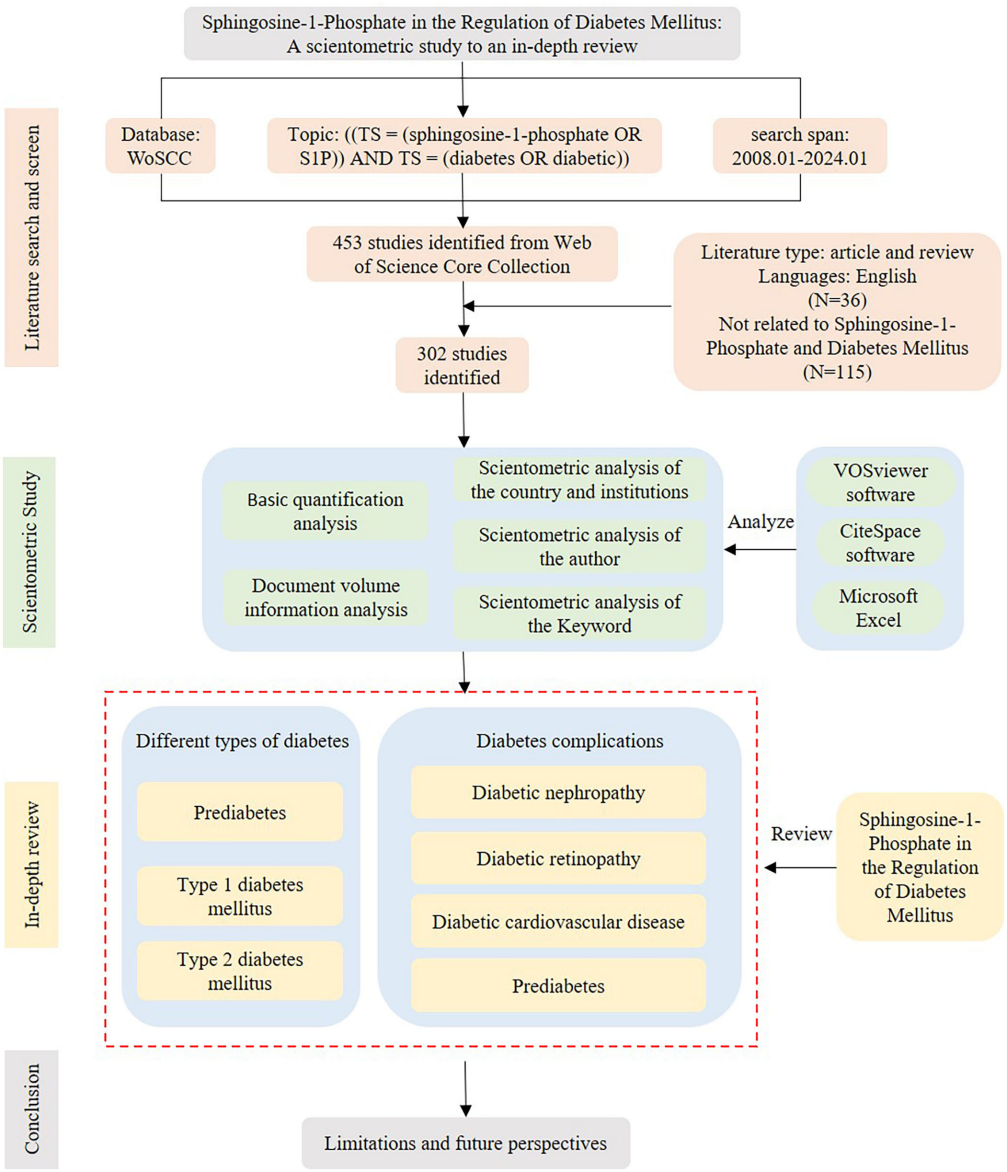
Research methodology. Sphingosine-1-Phosphate in the regulation of diabetes mellitus.

A comprehensive search was conducted in the Web of Science Core Collection (WoSCC) with the keywords: “TS = (Sphingosine-1-Phosphate OR S1P)” AND “TS = (diabetes OR diabetic)”. The research covered the period from January 2008 to January 2024, focusing specifically on English “Articles” and “Reviews”. The research methodology is illustrated in **Figure 1**.

### 2.2 Data analysis tools

We utilized VOSviewer (version 1.6.19), specifically designed for bibliometric analysis, to construct and comprehensively analyze bibliometric networks. Simultaneously, we employed CiteSpace (version 6.3.R1), a JAVA-based information visualization tool, to complement our analysis. These tools enable us to rigorously examine various dimensions, including countries, institutions, authors, and keyword co-occurrences, thus providing a detailed exploration of the structural and relational dynamics within the scientific network.

### 2.3 Basic quantification analysis

This research examines 302 papers, reflecting the collaboration of 1639 authors from 470 organizations in 37 countries, and published across 178 distinct journals. The papers in this analysis collectively reference 14,385 articles from 2097 journals, with 687 keywords surfacing throughout the study. This vast dataset highlights the global academic interconnectedness and the multidisciplinary aspect of this research.

### 2.4 Document volume information analysis

**Figure 2:**
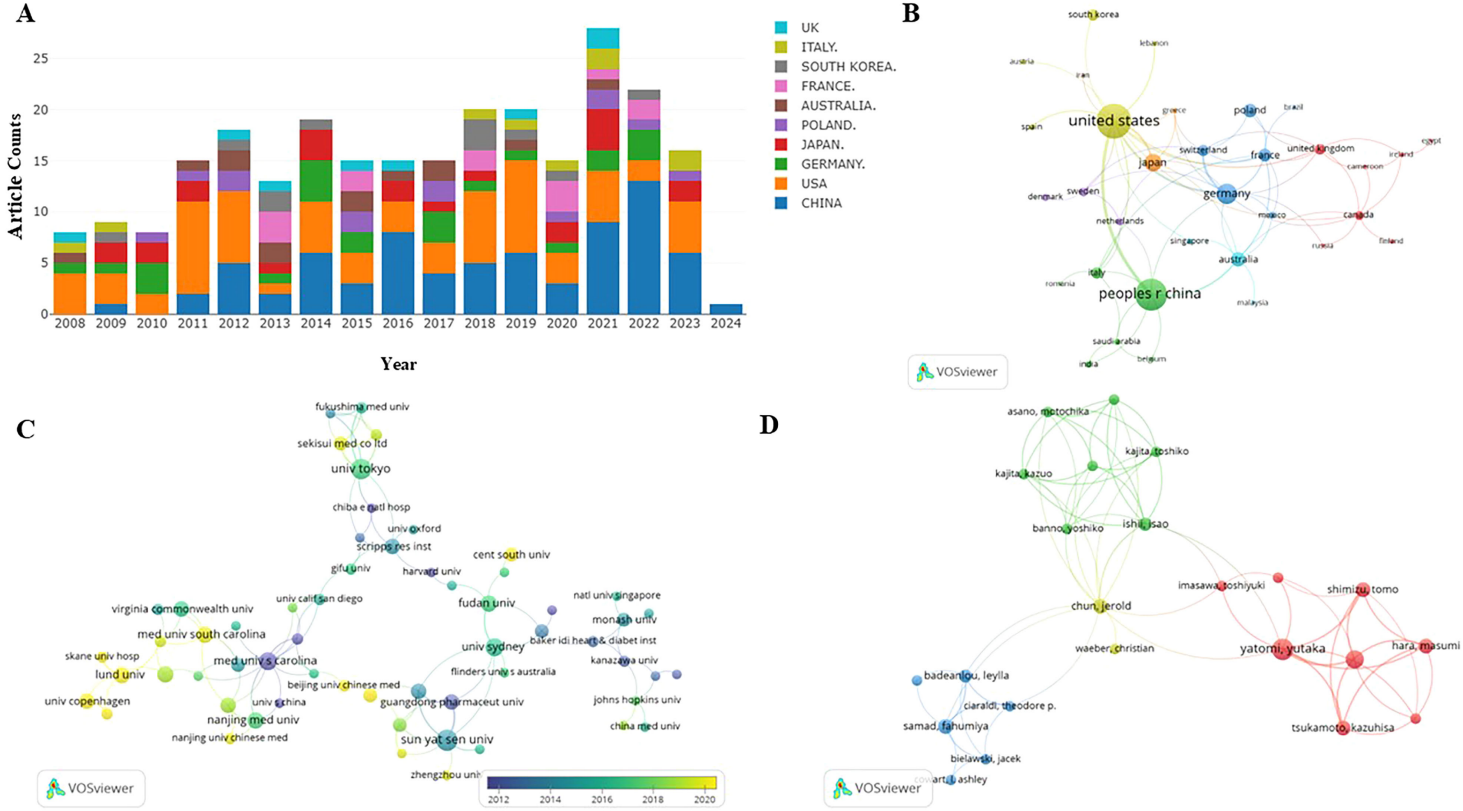
**(A)** S1P in diabetes mellitus distribution of publications. incomplete data for 2024. **(B)** Distribution and international cooperation of countries that are involved in S1P in the regulation of diabetes mellitus. **(C)** The visualization of institutions on the research of S1P in the regulation of diabetes mellitus. **(D)** The visualization of authors on research of S1P in the regulation of diabetes mellitus. Node size indicates the number of publications. Lines between nodes indicate the existence of a cooperative relationship; thicker lines indicate a closer relationship.

The quantity of publications visually reflects the developmental trends within a specific field over a designated period. **Figure 2A** depicts the yearly trends in publications concerning S1P research in diabetes. Generally, since 2008, there has been a consistent increase in research on S1P and diabetes, evidenced by a substantial rise in the annual publication count. The peak of publications occurred in 2021, with the release of 35 papers. While data for 2024 are still forthcoming, the trend in research publications is anticipated to maintain its upward trajectory. This suggests that research on S1P in the context of diabetes is increasingly becoming a focal point in this area.

### 2.5 Scientometric analysis of the country and institutions

This research evaluated publications from 37 countries or regions to pinpoint significant contributors to S1P research in diabetes regulation. As outlined in **Table 1**, the United States is at the forefront in terms of both the number of publications and citations, with China closely trailing, noted for its high publication volume and the significant total and average citations per publication. Furthermore, a collaborative network was developed, as shown in **Figure 2B**, depicting the interconnections between the number of publications and the participating countries.

**Table 1:** The top 10 countries/regions and Institutions contributing to publications about S1P in the regulation of diabetes mellitus.

Rank	Countris/ Regions	Article counts	Total citations	Average citations	Institutions	Article counts	Total citations	Average citations
1	United States	99	5041	50.91	Medical University of Bialystok	11	228	20.72
2	China	82	1697	20.69	Sun Yat-Sen University	10	329	32.9
3	Germany	34	900	26.47	University of Tokyo	9	238	26.44
4	Japan	26	631	24.26	Medical University of South Carolina	7	341	48.71
5	Australia	18	829	46.05	University of Bern	7	240	34.28
6	Poland	15	406	27.06	University of Sydney	7	271	38.71
7	France	14	325	23.21	University of Virginia	6	202	33.66
8	Switzerland	11	503	45.72	University of Miami	6	191	31.83
9	South Korea	11	341	31	Fudan University	6	124	20.66
10	Italy	11	254	23.09	Medizinische Hochschule Hannover	6	122	20.33

Similarly, this study pinpointed 470 institutions contributing significantly to the research on S1P in diabetes regulation. **Table 1** ranks the top ten institutions by number of publications, highlighting the Medical University of Bialystok, Sun Yat-Sen University, and the University of Tokyo as leaders. Significantly, the Medical University of South Carolina is distinguished by its high total and average citation counts. Visual analysis depicted in **Figure 2C** reveals 121 institutions with at least two publications, illustrating a network of collaboration that emphasizes publication volume and inter-institutional relationships. In this network, the size of circular nodes corresponds to the number of articles each institution has published, the thickness of lines between nodes denotes the frequency of collaboration, and the color of nodes indicates different clusters.

### 2.6 Scientometric analysis of the author

**Table 2** shows that the leading 10 authors together produced 60 articles on sphingosine-1-phosphate in diabetes treatment, accounting for 28.98% of the entire study, with each author contributing a minimum of 5 publications. Among the most prolific authors, Huang HQ stands out with 9 publications spanning from 2008 to 2024. Additionally, his publications have accumulated 319 citations, averaging 35.44 citations per publication. Remarkably, Huang KP, with only six publications, has achieved an impressive average citation rate of 45.50, underscoring the high quality of her research. **Figure 2D** illustrates the collaborative dynamics among authors, identifying four key teams that contribute to S1P research in diabetes regulation. However, limited cooperation and communication among these teams have led to suboptimal integration and utilization of resources.

**Table 2:** The top 10 authors contributing to publications about S1P in the regulation of diabetes mellitus.

Rank	Authors	Article counts	Total citations	Average citations
1	Huang, HQ	9	319	35.44
2	Liu, PQ	8	314	39.25
3	Pfeilschifter, J	8	246	30.75
4	Yatomi, Y	8	226	28.25
5	Huwiler, A	7	240	34.29
6	Huang, KP	6	273	45.50
7	Kurano, M	6	152	25.33
8	Hammad, SM	5	103	20.60
9	Lu, HW	5	101	20.20
10	Xiang, H	5	101	20.20

### 2.7 Scientometric analysis of the keyword

**Figure 3:**
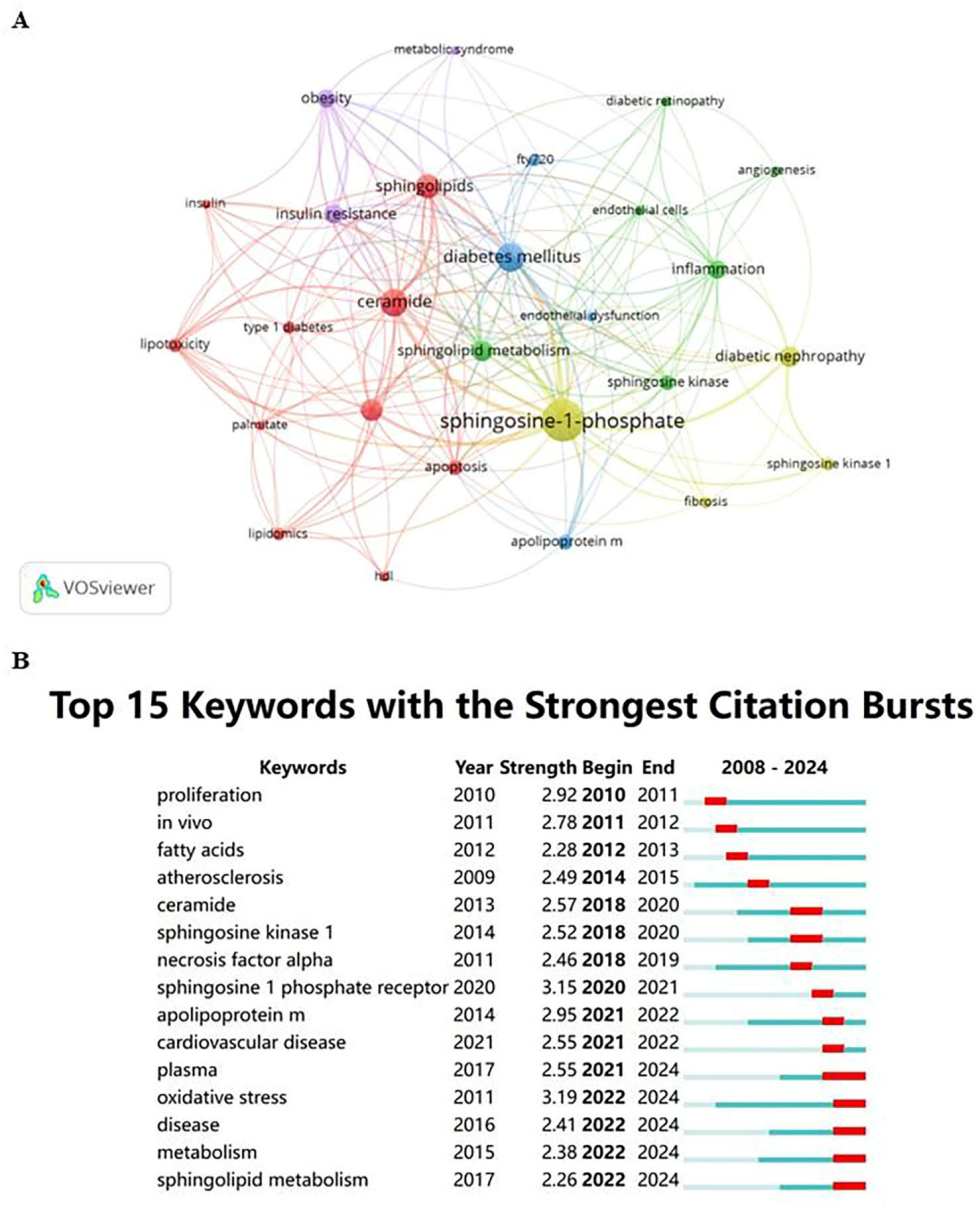
**(A)** Cluster visualization for keywords. Note: Nodes represent keywords; the size of the nodes indicates the frequency of keyword occurrence, with larger nodes indicating higher frequencies; the color of the nodes represents the cluster to which the keyword belongs; lines between nodes indicate co-occurrence relationships, with thicker lines representing stronger relationships. **(B)** Citation bursts of the top 15 keywords. Note: Light blue bars indicate the period before the references appeared, dark blue bars indicate when the references began to appear, and red bars represent the burst period. The longer the red bar, the longer the duration of the reference’s prominence.

Keywords are essential in any article as they highlight research topics and reveal trends in specific areas via co-occurrence analysis. Utilizing VOSviewer, 27 out of 687 keywords, each occurring at least five times, were chosen for co-occurrence analysis to pinpoint research hotspots. **Figure 3A** illustrates five key research directions, each represented by a distinct cluster:

#### 2.7.1 Biochemical and metabolic processes of diabetes

This cluster focuses on the biochemistry and metabolism of diabetes, encompassing key terms like apoptosis, ceramide, HDL, insulin, lipidomics, lipotoxicity, palmitate, sphingolipids, and both types of diabetes mellitus. It highlights the crucial role of metabolic disorders and lipid-mediated effects in diabetes.

#### 2.7.2 Angiogenesis and inflammatory mechanisms in diabetic complications

This cluster centers on angiogenesis and inflammation, featuring keywords such as angiogenesis, diabetic retinopathy, endothelial cells, inflammation, sphingolipid metabolism, and sphingosine kinase, indicating the vascular and inflammatory aspects of complications.

#### 2.7.3 Diabetes treatment and management strategies

It covers treatment and management strategies with terms like apolipoprotein M, diabetes mellitus, endothelial dysfunction, and FTY720, emphasizing disease management and therapeutic approaches.

#### 2.7.4 Targeted research on specific complications

Focuses on biomarkers and therapeutic targets for specific complications, including diabetic nephropathy, fibrosis, sphingosine kinase 1, and sphingosine-1-phosphate.

#### 2.7.5 The role of obesity and metabolic syndrome in diabetes

Highlights the interplay between obesity, metabolic syndrome, and diabetes with keywords such as insulin resistance, obesity, and metabolic syndrome, stressing the significant role of obesity and insulin resistance in the pathology of the disease.

These clusters help delineate the scope of current research and likely future directions in the study of Sphingosine-1-Phosphate and its impact on diabetes regulation.

Keyword burst analysis was carried out to pinpoint frequently cited emerging concepts across specific time intervals. In the burst analysis illustrated in **Figure 3B**, intervals and durations of citation bursts are indicated in blue and red, respectively. From 2020 to 2024, “oxidative stress” and “sphingosine 1 phosphate receptor” registered the highest burst intensities, scoring 3.19 and 3.15 respectively, establishing them as pivotal research hotspots.

Furthermore, the abstracts and complete texts of 302 articles were meticulously analyzed to investigate the roles of SIP in prediabetes, type 1 diabetes, type 2 diabetes, and associated complications like diabetic nephropathy, retinopathy, cardiovascular disease, and neuropathy. These results uncover fundamental research questions and offer insights into upcoming research directions and trends. A thorough review was conducted to deepen knowledge of S1P’s impact on diabetes, as depicted in the research methodology in **Figure 1**.

## 3 Different types of diabetes

S1P is produced by the deacylation of ceramide via ceramidase, leading to the formation of sphingosine. Following this, sphingosine kinase (SphK) phosphorylates sphingosine, producing S1P. This pathway includes two isoforms of SphK, SphK1 and SphK2 (21), which are widely expressed. With new drugs targeting S1P receptors or kinases, publications on S1P have increased exponentially. Studies show that alterations in the S1P axis, including its production, transport, and receptors, affect metabolism and cellular signaling, thus impacting different stages of diabetes progression.

### 3.1 Prediabetes

**Figure 4:**
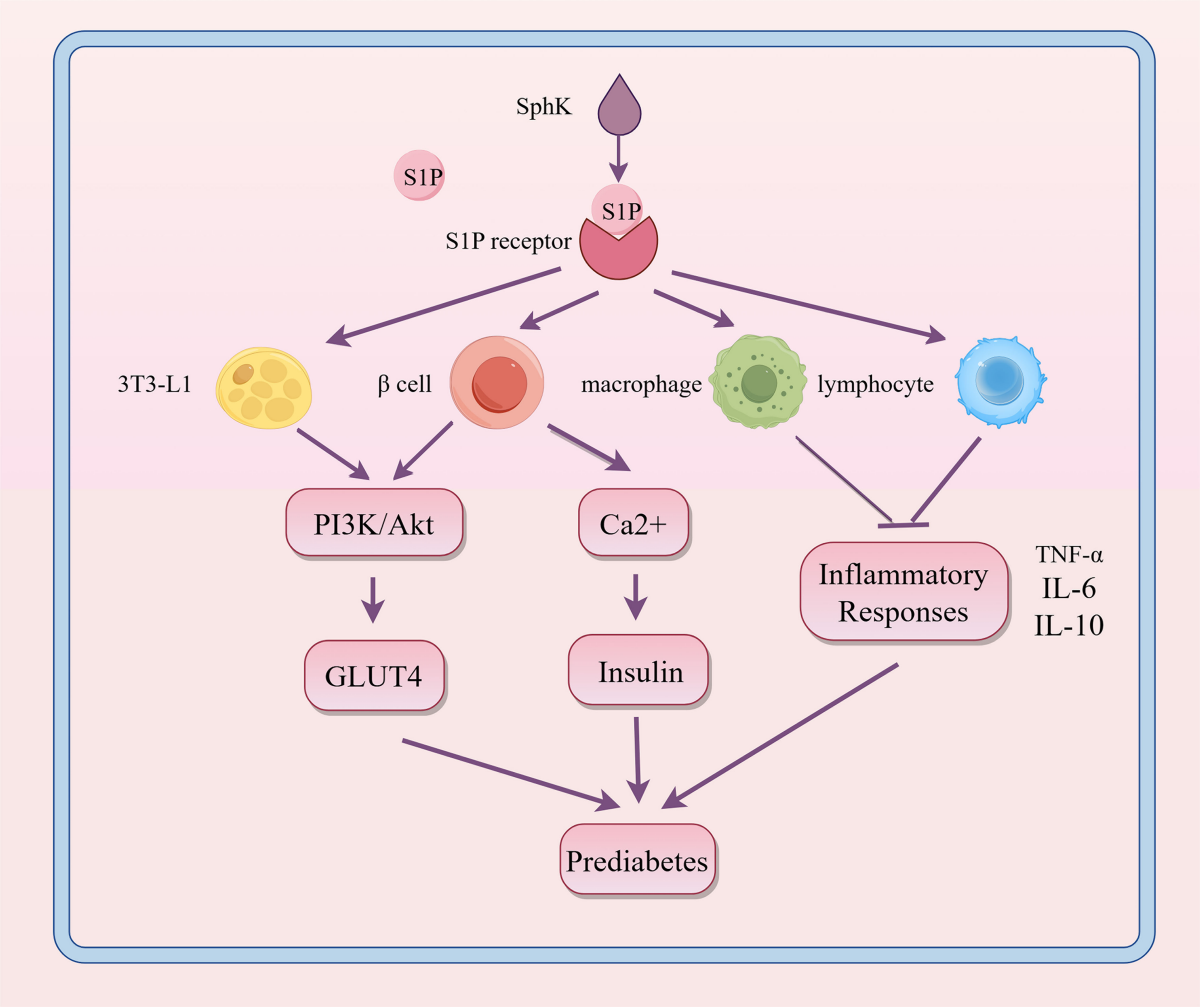
Regulatory role of S1P signaling in the progression of prediabetes. S1P interacts with its receptors on various cell types, influencing metabolic and inflammatory pathways. This interaction enhances PI3K/Akt-mediated GLUT4 translocation and boosts Ca2+ levels in β cells, thereby promoting insulin secretion. It also modulates inflammatory responses in macrophages and lymphocytes. These interactions help maintain glucose homeostasis and regulate inflammation, which are crucial in the development of prediabetes. SphK, sphingosine kinase; S1P, sphingosine-1-phosphate; PI3K, phosphoinositide 3-kinase; Akt, protein kinase B; GLUT4, glucose transporter type 4; TNF-α, tumor necrosis factor alpha; IL-6, interleukin 6; IL-10, interleukin 10.

Obesity serves as a critical pathological contributor to prediabetes. Adipose tissue (AT) is pivotal in the development of obesity-related complications. In adipose tissue, inflammation is propelled by enlarged adipocytes and worsened by the infiltration of adipose tissue macrophages (ATM) and other immune cells. Impaired adipose tissue promotes ectopic lipid deposition in organs such as the liver, skeletal muscle, and pancreas, leading to lipotoxicity, metabolic disturbances, and accelerated progression of insulin resistance (IR) and diabetes (22, 23). Previous research indicates that S1P might influence obesity or metabolic syndrome by altering lipid metabolism, insulin sensitivity, and inflammatory responses, thereby playing a role in the onset of prediabetes, as shown in **Figure 4**.

Increased levels of S1P in tissues and plasma are identified as a significant marker of obesity in both humans and rodents, suggesting a role for S1P metabolism in the development of T2DM (24). In a cohort study involving 1339 healthy individuals, Moritz, E. et al. found that the median serum S1P levels were 0.804 (0.694; 0.920) μmol/L, significantly higher than in individuals exhibiting metabolic abnormalities (25). S1P also controls glycogenolysis and gluconeogenesis, thereby affecting glucose homeostasis by facilitating increased glucose uptake in mouse embryonic fibroblast (preadipocyte) line (3T3-L1) adipocytes (26). Studies show that obesity increases SphK1 expression in adipose tissue macrophages of both M1 and M2 phenotypes (27). SphK1 affects the migration and activation of inflammatory cells like macrophages and lymphocytes, as well as the production of cytokines including tumor necrosis factor-alpha (TNF-α), interleukin-6 (IL-6), and IL-10, thus serving both pro-inflammatory and anti-inflammatory functions in obesity (28). Moreover, in the initial stages of prediabetes and type 1 diabetes, the S1P/S1PR axis may play a protective role by influencing the survival and anti-inflammatory impacts on pancreatic β-cells. For instance, Japtok et al. showed that the S1P receptor antagonist JTE-013 effectively reduced β-cell damage, highlighting the essential role of S1P(2) in maintaining β-cell homeostasis (29).

In conclusion, the role of S1P in regulating lipid metabolism has become a critical focus in metabolic disease research. By affecting lipid metabolism, insulin sensitivity, and inflammatory responses, S1P impacts risk factors for prediabetes and accelerates the shift from prediabetes to diabetes. These complex interactions highlight S1P’s significance as a potential therapeutic target, especially in managing metabolic syndrome and conditions leading up to diabetes. Investigating the specific mechanisms and pathways affected by S1P can aid in developing potential treatment strategies.

### 3.2 Type 1 diabetes mellitus

**Figure 5:**
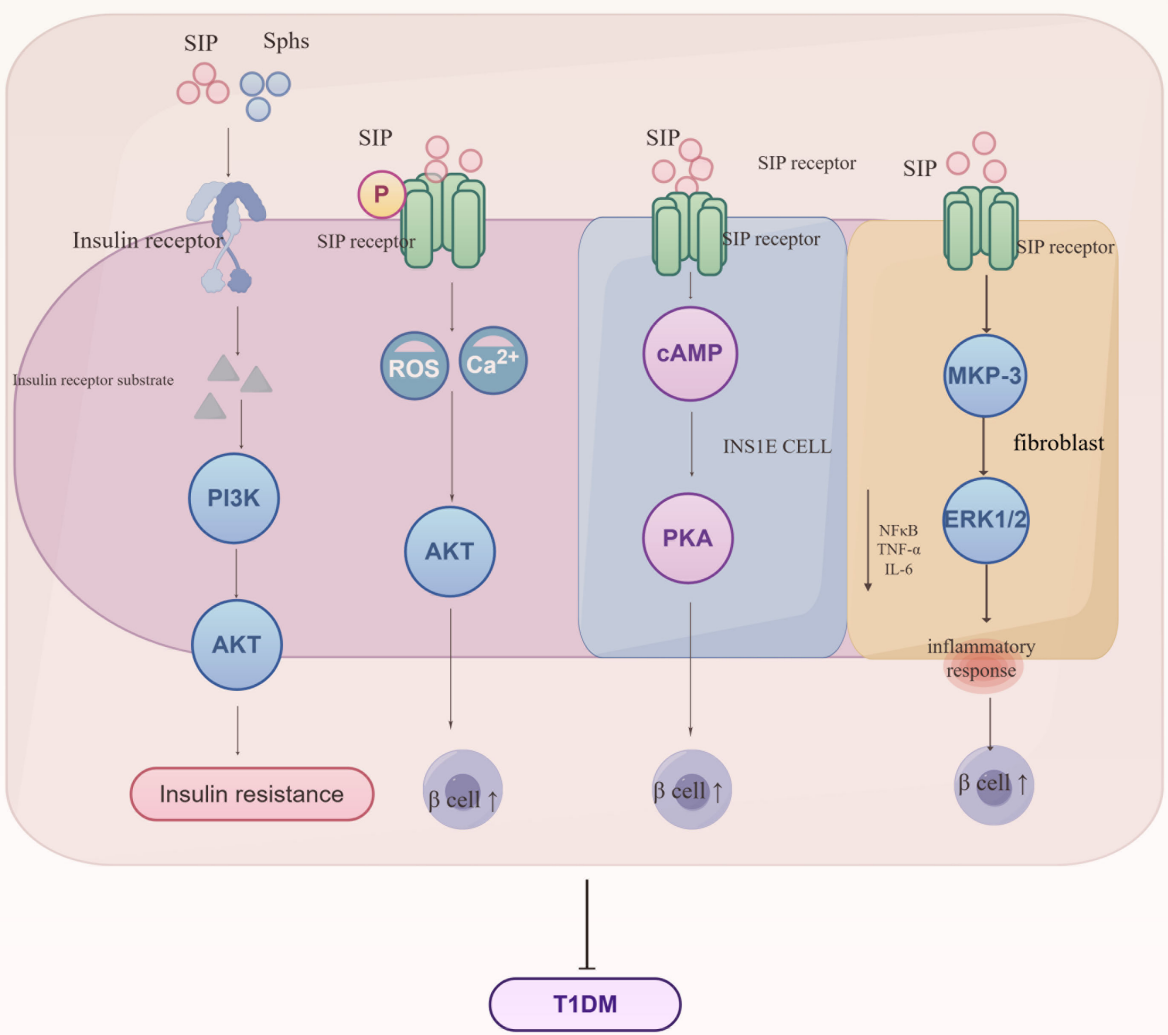
Regulatory mechanisms of S1P signaling in the progression of Type 1 Diabetes Mellitus (T1DM). S1P interacts with insulin receptors to influence the synthesis of insulin receptor substrates, subsequently mediating the PI3K/AKT signaling pathway and leading to insulin resistance. Additionally, S1P interacts with receptors on various cell types, regulating metabolic and inflammatory pathways crucial for the development of insulin resistance and T1DM. S1P signaling enhances Ca2+ influx and reactive oxygen species (ROS) generation, thereby impacting AKT activity and β-cell function. In INSIE cells and fibroblasts, S1P affects cAMP levels and the MAPK pathway, influencing PKA activity and inflammatory responses mediated by MKP-3, ERK1/2, NF-κB, TNF-α, and IL-6. These interactions collectively promote the regulation of glucose homeostasis and inflammation, key processes in the pathogenesis of T1DM. Sphs, sphingosines; SIP, sphingosine-1-phosphate; PI3K, phosphoinositide 3-kinase; AKT, protein kinase B; cAMP, cyclic adenosine monophosphate; PKA, protein kinase A; MKP-3, MAP kinase phosphatase-3; ERK1/2, extracellular signal-regulated kinase 1/2; NF-κB, nuclear factor kappa-light-chain-enhancer of activated B cells; TNF-α, tumor necrosis factor alpha; IL-6, interleukin 6.

Type 1 diabetes mellitus generally occurs when the immune system erroneously attacks and destroys the insulin-producing β-cells in the pancreas, significantly reducing or completely halting insulin production (30). Globally, Type 1 diabetes is rare, typically emerging in childhood or early adolescence but can appear at any age, requiring lifelong insulin therapy to maintain blood sugar levels (31). For those diagnosed and their families, this condition is serious and long-term, requiring ongoing management, as shown in **Figure 5**.

Sphingolipids and S1P indirectly impact the PI3K/Akt signaling pathway by altering insulin receptor autophosphorylation and insulin receptor substrate (IRS) protein activity, which weakens insulin signaling and contributes to insulin resistance development. Studies show that S1P controls insulin production by modulating intracellular calcium levels and reactive oxygen (ROS) generation, enhancing β-cell survival and reducing apoptosis through activation of the Akt pathway, potentially providing early-stage disease protection (32). However, this protective benefit decreases as the disease advances. Additional studies demonstrate that S1P boosts cyclic adenosine monophosphate (cAMP) production in INS1E cells, safeguarding β-cells via protein kinase A (PKA) activation and regulation of calcium homeostasis (33). NOD (non-obese diabetic) mice inherently develop type 1 diabetes via T-cell-mediated destruction of pancreatic cells. Studies indicate that NOD mice exhibit decreased S1P1 and sphingosine-1-phosphate lyase (SGPL1) expression in thymocytes, along with T-cell migration abnormalities, implying that regulation of S1P1 and its interactions in thymocytes may play a role in the onset of type 1 diabetes (34). Furthermore, S1P significantly increases MKP-3 levels in fibroblasts, boosts ERK1/2 phosphorylation, and diminishes NF-κB activation, thus reducing inflammatory responses in endothelial cells (35). S1P protects insulin-secreting INS1E cells and rat islets against cytokine toxicity. During the acute phase of cytokine toxicity, the expression of SK2, S1P transporters, and receptors is upregulated.

In conclusion, S1P boosts β-cell survival by reducing inflammatory factors, thus providing protection against type 1 diabetes.

### 3.3 Type 2 diabetes mellitus

**Figure 6:**
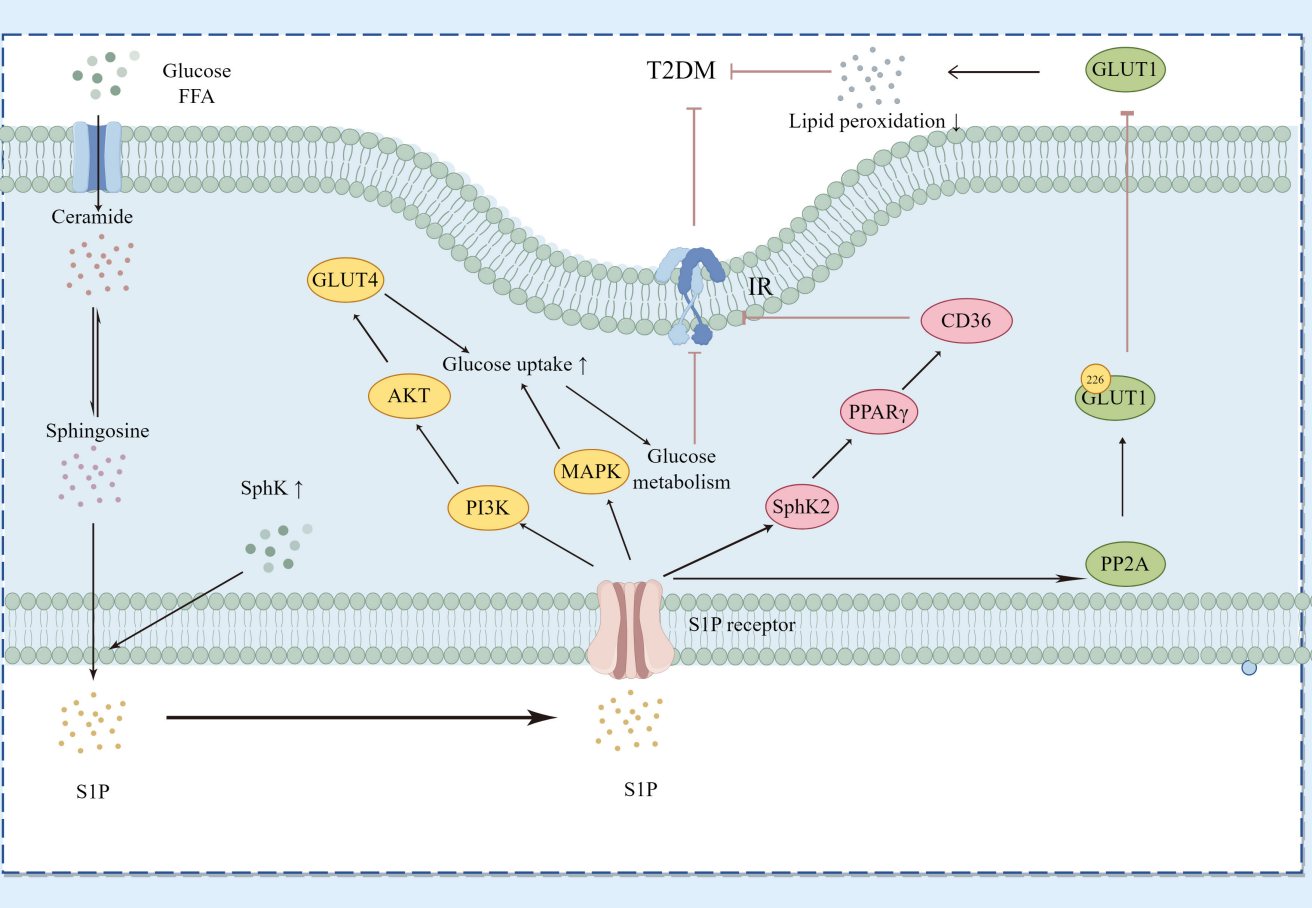
Regulatory Pathways of Sphingosine-1-Phosphate (S1P) in Type 2 Diabetes Mellitus (T2DM). This diagram demonstrates how S1P interacts with specific receptors on various cell types to influence glucose and lipid metabolism, thereby affecting key signaling pathways associated with T2DM. S1P modulates the PI3K/Akt pathway to regulate GLUT4 translocation and the MAPK pathway to impact glucose uptake and metabolism. Additionally, it alters insulin receptor signaling by influencing ceramide and sphingosine levels, thus impacting insulin resistance. S1P, through SphK2, affects the expression levels of PPARγ and its downstream protein CD36, improving sugar and lipid metabolism as well as insulin resistance. Moreover, S1P activates PP2A to inhibit the translocation of GLUT1 on the cell surface, reducing lipid peroxidation. The dynamic balance between SphK1 and SphK2 enzyme activities determines the overall levels of S1P, linking sphingolipid metabolism with insulin resistance and inflammation in T2DM. Key components include Sphs (sphingosines), S1P, GLUT4, GLUT1, PI3K (phosphoinositide 3-kinase), AKT (protein kinase B), MAPK (mitogen-activated protein kinase), PPARγ (peroxisome proliferator-activated receptor gamma), CD36 (cluster of differentiation 36), and PP2A (protein phosphatase 2A).

T2DM is the most common form of diabetes globally, with its incidence rapidly growing due to increasing obesity rates and an aging worldwide population (36). Characterized by insulin resistance and a relative insulin deficiency, T2DM often co-occurs with obesity, metabolic syndrome, and chronic inflammation (37), as shown in **Figure 6**.

In T2DM, the S1P/S1PR axis influences disease progression by controlling insulin signaling pathways, lipid metabolism, and inflammatory responses. Continuous hyperglycemia and lipid dysregulation can alter S1P signaling, exacerbating insulin resistance and beta-cell dysfunction via changes in S1P receptor expression or the activity of downstream signaling molecules. SphK1 facilitates the transformation of ceramide into S1P, thereby enhancing insulin sensitivity (38). S1P interacts with its specific G-protein-coupled receptors (S1PRs) and triggers important downstream insulin signaling pathways such as PI3K/Akt and MAPK. The insulin signaling pathway in skeletal muscle centers on PI3K/Akt, promoting glucose uptake, utilization, and storage mediated by Akt through GLUT4 modulation. Overexpression of SphK1 markedly enhances hepatic insulin signaling and glucose tolerance in KK/Ay diabetic mice (39). Elevated SphK2 protein expression in the S1P/Cer signaling pathway inhibits PPARγ and its downstream CD36 protein, thereby improving hepatic glucose and lipid metabolism and reducing insulin resistance (40). Higher levels of S1P and enhanced activity of PP2A have been observed in red blood cells from patients with T2DM and mice with chronic hyperglycemia. S1P activates PP2A, which dephosphorylates serine 226 in GLUT1, obstructing GLUT1’s movement to the cell surface and reducing glucose uptake in diabetic mice and human erythrocytes, thereby protecting against lipid peroxidation under hyperglycemic and diabetic conditions. This process might function in insulin-independent tissues like the brain, meriting additional research (41). While T2DM is primarily characterized by insulin resistance, the decline in beta-cell function also greatly contributes to disease progression. S1P may protect beta-cells from high-fat, high-sugar environments, aiding in the maintenance of normal insulin secretion. Extracellular S1P encourages beta-cell proliferation and reduces apoptosis in diabetic mice induced by HFD/STZ (42).

Lipid metabolism and inflammatory processes are essential in the development of T2DM. S1P impacts metabolic status and insulin resistance through the regulation of lipolysis, fatty acid release, and activation of inflammatory cells. The S1P-S1PR3 signaling pathway promotes adipogenesis and shows anti-inflammatory effects in adipose tissue, while also demonstrating anti-inflammatory and anti-steatotic properties in the liver (43). Cells release active NCDase through exosomes, protecting against FFA-induced apoptosis by modifying sphingolipid metabolites, potentially offering a therapeutic approach for cellular lipotoxicity and T2DM. Increased expression of SphK1 inhibits disruptions in protein transport from the endoplasmic reticulum to the Golgi, mediated by palmitate, and protects beta-cells from lipotoxicity *in vivo (* 44). Additionally, heightened expression of SphK1 inhibits lipotoxicity induced by palmitate in INS-1 cells (45).

In summary, S1P plays multiple roles in both type 1 and type 2 diabetes, significantly affecting immune modulation and beta-cell protection in type 1 diabetes, and more profoundly influencing insulin sensitivity, lipid metabolism, and inflammatory responses in type 2 diabetes. Understanding these mechanisms is crucial for developing innovative diabetes treatment strategies that focus on the S1P pathway.

## 4 Diabetes complications

**Figure 7:**
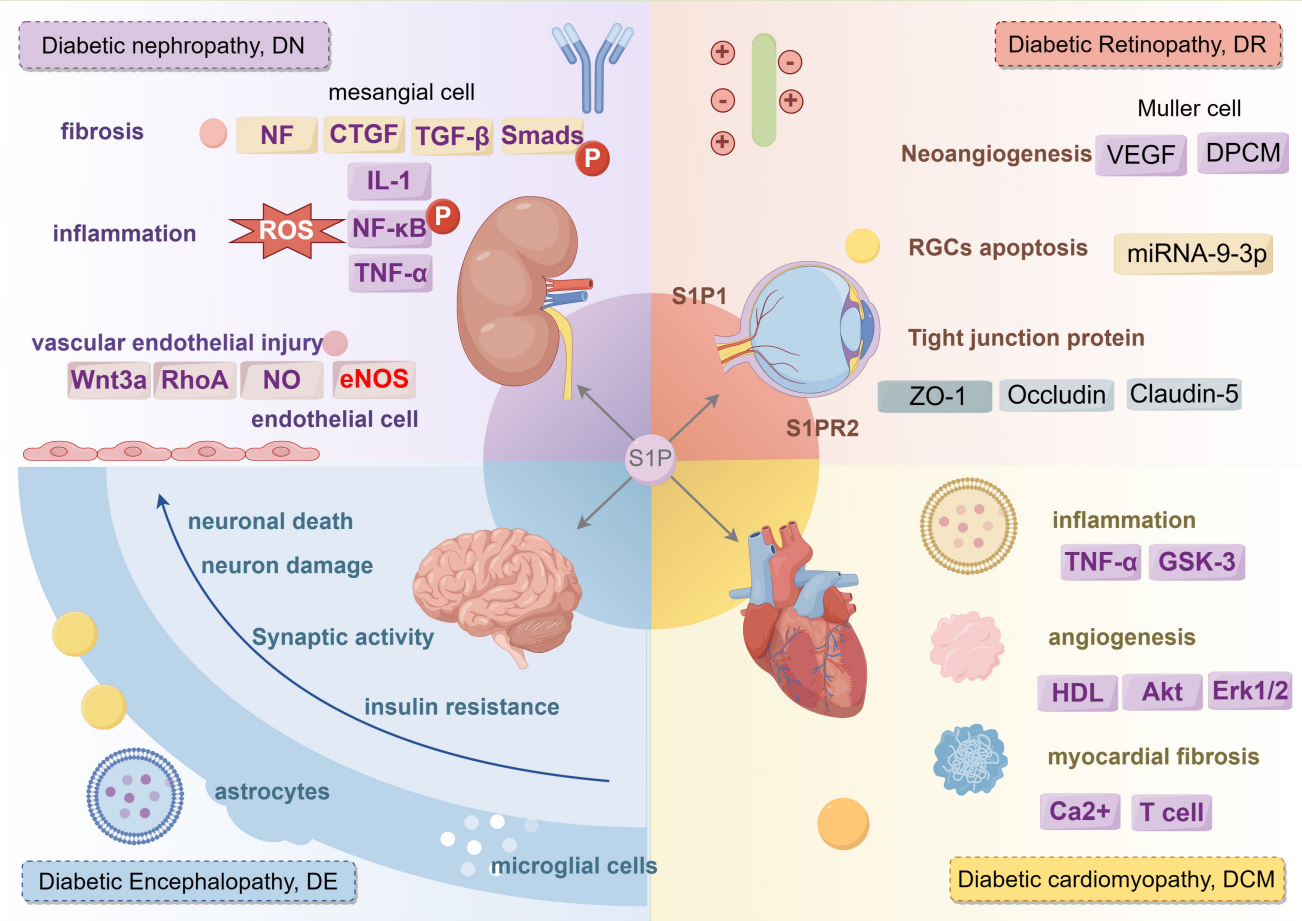
The regulatory role of S1P signaling in different complications of diabetes mellitus. S1P mainly plays a role in diabetic nephropathy from three aspects: fibrosis, inflammation and vascular endothelial injury. S1P plays a role in diabetic retina from three aspects: Neoangiogenesis, RGCs cell apoptosis and tight junction protein. S1P promotes diabetic cardiovascular disease from three aspects: inflammation, angiogenesis, myocardial fibrosis. S1P affects diabetic peripheral neuropathy from neuronal death, neuron damage, synaptic activity, insuline resistance. IL-1, Interleukin-1; CTGF, connective tissue growth factor; TGF-β, transforming growth factor-β; NF-κB, nuclear factor kappa-B; TNF-α, tumor necrosis factor-α; GSK-3, glycogen synthase kinase-3; Akt, Protein kinase B; ERK1/2, extracellular regulated protein kinase.

Clinically, diabetes is linked with a variety of complications. S1P not only affects different types of diabetes but also plays a role in complications such as diabetic nephropathy, diabetic retinopathy, diabetic cardiovascular disease, and diabetic neuropathy, as shown in **Figure 7**.

### 4.1 Diabetic nephropathy

Diabetic nephropathy (DN), also known as diabetic kidney disease (DKD), ranks as one of the most severe chronic complications of diabetes, frequently leading to renal failure, dialysis, kidney transplants, and end-stage renal disease (ESRD). Extended hyperglycemia causes chronic inflammation and metabolic disruptions, ultimately leading to long-term damage to the structure and function of renal cells (46). Research indicates that S1P might affect the progression of diabetic nephropathy by influencing fibrosis, inflammation, immune responses, and vascular endothelial damage in the kidneys.

S1P promotes the transformation of fibroblasts and renal tubular epithelial cells (EMT) in the kidney, a crucial phase in the fibrosis process. Research shows that S1P engages with the transforming growth factor (TGF)-β signaling pathway through specific receptors, thereby stimulating fibroblast activation and proliferation, and enhancing the production of extracellular matrix proteins like collagen and fibronectin. These changes ultimately result in alterations in kidney structure and loss of function, pivotal in the progression of diabetic nephropathy (47). TGF-β2 induces connective tissue growth factor (CTGF) expression in several cell types, including human mesangial cells, emphasizing the role of S1P in mesangial cell fibrosis in diabetic nephropathy. Extracellular S1P advances renal fibrosis through activation of cell surface S1P receptors, whereas intracellular S1P reduces fibrosis, potentially connected to the SphK1 and S1P pathways (48). Clinical studies show that serum apolipoprotein M (ApoM) levels in patients with type 2 diabetes inversely correlate with the progression of diabetic nephropathy, suggesting that ApoM/S1P could help in preventing and mitigating diabetic nephropathy. The ApoM/S1P axis diminishes Smad3 pathway activation and enhances nitric oxide synthase (eNOS) phosphorylation via the S1P1 pathway (46). Furthermore, ApoM/S1P raises SIRT1 protein levels, boosts mitochondrial function, and could lead to selective S1P1 activation by elevating cellular membrane S1P content. ApoM could act as a valuable biomarker for predicting the progression of diabetic nephropathy, and the ApoM/S1P–S1P1 axis might emerge as a new therapeutic target for its prevention and treatment (49).

In diabetic nephropathy, S1P could intensify inflammatory kidney damage by modulating the activity of inflammatory cells and the secretion of pro-inflammatory cytokines. Studies indicate that S1P controls key inflammatory signaling pathways, such as NF-κB, influencing the migration and activation of inflammatory cells like macrophages and T cells, as well as the balance between pro- and anti-inflammatory cytokines.

S1P controls vascular dilation and constriction, affecting blood flow and pressure, which ultimately leads to renal microvascular damage. S1P also impacts renal cell survival and apoptosis; an imbalance can lead to substantial renal cell damage and apoptosis, thus accelerating the progression of diabetic nephropathy (50, 51). Bekpinar et al. found that decreased plasma S1P levels might adversely affect endothelial integrity and barrier function in patients with diabetic nephropathy, potentially triggering a detrimental cycle (52).

Although substantial research has been conducted on the role of S1P in diabetic nephropathy, additional studies are required to completely clarify its mechanisms and therapeutic possibilities. Future investigations should focus on precisely modulating S1P signaling to reduce the progression of diabetic nephropathy and improve patient outcomes.

### 4.2 Diabetic retinopathy

Diabetic retinopathy (DR) is the most common and serious eye-related complication of diabetes and a leading cause of blindness among the global working-age population. Approximately one-third of individuals with diabetes are estimated to experience some form of retinopathy (53). Chronic hyperglycemia in diabetes can lead to microvascular damage, crucial for the development of diabetic retinopathy. Microvascular damage leads to increased vascular permeability, vessel occlusion, and affects neovascularization (54). Hyperglycemia-induced microvascular damage reduces blood supply to the retina, resulting in hypoxia. Hypoxia then activates the expression of growth factors such as vascular endothelial growth factor (VEGF), which encourages neovascularization. These newly developed blood vessels are delicate and susceptible to rupture, resulting in bleeding, retinal edema, and eventually vision loss.

In diabetic retinopathy, S1P facilitates abnormal blood vessel formation by affecting critical factors such as VEGF. S1P controls the inflammatory response and apoptosis in retinal nerve cells and vascular endothelial cells, significantly impacting their proliferation, migration, and lumen formation. By altering signaling pathways such as NF-κB and caspase, S1P impacts the production of proinflammatory cytokines and apoptosis, thus intensifying retinopathy. Clinical research has demonstrated that average S1P levels in the aqueous humor of patients with proliferative diabetic retinopathy are significantly higher, and elevated serum S1P levels are also observed in diabetic patients (55). A different clinical study showed that S1PR1 expression in vitreous samples from patients with proliferative diabetic retinopathy is notably higher than in the non-diabetic control group, underscoring the vital role of sphingolipid metabolism in the progression of diabetic retinopathy (55, 56). S1P can protect against the degeneration of photoreceptors and ganglion cells, yet it also promotes inflammation, fibrosis, and neovascularization in conditions like AMD, glaucoma, and pro-fibrotic diseases. Inhibitors that regulate S1P signaling, including myricetin, desipramine, and fingolimod (FTY720), have demonstrated effectiveness in preserving neuronal viability and retinal function (57).

Research indicates that adjusting sphingolipid rheostats could potentially serve as a treatment for retinal degenerative diseases. Diabetes impacts pericyte contractility and cytoskeletal signaling pathways. The use of S1P receptor antagonists can reduce DPCM-induced growth in retinal endothelial cells and S1P-mediated pericyte contraction, thus affecting retinal endothelial proliferation and vascularization (58). Studies show that the proliferation and migration of retinal Muller glial cells play roles in the pathology of diabetic retinopathy, and S1P can prompt Muller cell migration via G protein-coupled receptor signaling, possibly enhancing diabetic retinopathy outcomes (59). Both *in vitro* and *in vivo* studies have verified that exosomes from Muller glia worsen vascular dysfunction under high glucose conditions. Mechanistically, exosomal miRNA-9-3p is conveyed to retinal endothelial cells, attaching to the coding sequence of the S1P(1) receptor, potentially providing new biomarkers for treating diabetic retinopathy (60). Notably, Sphingosine-1-Phosphate (S1P) also exhibits dual roles in diabetic retinopathy. The action of S1P on the S1P(1) receptor can reduce angiogenesis, increase endothelial integrity, decrease photoreceptor cell apoptosis, and protect the retina from neurodegeneration.

In contrast, signaling through the S1P(2) receptor can encourage neovascularization, impair endothelial connections, trigger VEGF release, and result in retinal cell apoptosis and neuroretinal degeneration (61). Glutamate excitotoxicity is identified as a contributing factor to diabetic retinopathy, and research has demonstrated that balancing the sphingolipid rheostat between S1P and ceramide contributes to neuroprotection, preventing excitotoxic RGC death (62). Consequently, modulating sphingolipid rheostats could potentially act as a therapeutic strategy for retinal degenerative diseases.

Exenatide can enhance recovery from HG-induced damage in human retinal vascular endothelial cells (hRVECs) by influencing S1PR2 production. Sphingosine-1 can negate the effects of exenatide, reducing ROS and apoptosis induced by high glucose. Targeting S1P modulation or inhibiting S1PR2 may provide therapeutic advantages for diabetes and safeguard against retinal neurodegeneration (63). FTY720 can inhibit the breakdown of the blood-retinal barrier (BRB) and prevent the reduction of tight junction proteins (ZO-1, Occludin, and Claudin-5) in diabetic rat retinas. Additionally, FTY720 can counteract the downregulation of S1P1 and S1P3 in these retinas, providing protection against diabetic retdinopathy (DR), likely due to its anti-inflammatory and barrier-enhancing effects. Modulating S1PR could represent a novel approach for treating diabetic retinopathy (64).

While the role of S1P in diabetic retinopathy is somewhat understood, numerous specific aspects still require further exploration. Future research is anticipated to elucidate the roles of S1P and its receptor subtypes in angiogenesis, inflammation, apoptosis, and fibrosis, potentially paving the way for novel therapeutic and preventive strategies for diabetic retinopathy.

### 4.3 Diabetic cardiovascular disease

Dyslipidemia and lipoprotein dysfunction are recognized as factors that heighten the risk of cardiovascular diseases. In diabetic individuals, glycation lowers the S1P content in HDL, and the S1P level in diabetic high-density lipoprotein inversely correlates with hemoglobin A1c (*P*<0.005) (65). Glycated HDL’s ability to activate protective intracellular survival pathways such as Akt, Stat3, and Erk1/2 is significantly reduced. Glycation lowers the S1P content in high density lipoprotein (HDL), leading to increased cardiomyocyte death. However, studies have shown that advanced glycation end products (AGEs) raise S1P levels in cells, and their inhibitors could prevent the development of diabetic cardiomyopathy (66). S1P exhibits multiple effects, including promoting cell proliferation, either inhibiting or stimulating cell migration, and regulating vasoconstriction or the release of vasoactive substances.

Diabetes might compromise the bioactive metabolites of S1P, potentially explaining the diminished cardioprotective effect of ischemic preconditioning (IPC) in diabetic hearts. The S1P agonist FTY720 notably decreases TNF-α and GSK-3 levels, and the release of LDH and CK-MB. FTY720 could potentially mitigate diabetic cardiac ischemic reperfusion injury by suppressing GSK-3 and modulating the opening of mitochondrial permeability transition pores (67). Additionally, FTY720 activates focal adhesion kinase-related adherens junctions (AJ) at intercellular contacts, coinciding with the formation of distinct cortical actin rings. Under both normal and high glucose conditions, FTY720 enhances trans-endothelial electrical resistance (TER) in HMVEC monolayers, suggesting strengthened endothelial barriers (68).

T-cell S1P1 signaling activation plays a dual role in the progression of cardiac fibrosis, affected by glucose levels: it exhibits antifibrotic properties under normal glucose conditions and intensifies fibrosis under elevated glucose levels. Targeted deletion of the T-cell S1P receptor 1 has been shown to alleviate cardiac fibrosis in streptozotocin-induced diabetic mice (69). S1P induces a rise in intracellular Ca2+ concentrations in various cell types. In type 2 diabetes, reduced S1P levels, heightened CD3(+)CD4(+)CD69(+) T-cell levels, and increased hsCRP, 2hPG, LDL, and Lp(a) levels all contribute to an elevated risk of coronary heart disease, highlighting the significant impact of S1P and immune cell dynamics on cardiovascular health in diabetic conditions (70). Sphingosine kinases 1 and 2 (SK1 and SK2) facilitate the transformation of the sphingolipid metabolite sphingosine into S1P. The balance between S1P levels and its precursors, ceramide and sphingosine, acts as a crucial switch affecting cellular proliferation or death. This balance, known as the “sphingolipid rheostat,” is primarily regulated by sphingosine kinases (SKs) and plays a role in vascular calcification.

In conclusion, the S1P/S1PR axis substantially impacts the progression of diabetic complications by influencing the function of blood vessels, nerves, and other tissues. For example, by modulating MAPK and Akt signaling in endothelial cells, S1P influences vascular inflammation and neovascularization, thus impacting microvascular complications in diabetes.

### 4.4 S1P and diabetic neuropathy

Diabetic neuropathy, a common complication of diabetes, manifests with a range of symptoms that depend on the type and extent of nerve damage. These symptoms typically include pain, numbness in the extremities, weakness, dizziness, gastroparesis, erectile dysfunction in males, and diabetic foot complications.

Cognitive dysfunction associated with diabetes, often referred to as diabetic encephalopathy, is a significant complication of the disease. Recent research shows that neuronal damage, neuroinflammation, and insulin resistance are key mechanisms that increase the risk of cognitive impairment in diabetes. Diabetes causes a reduction of ceramide in the hippocampus and cerebellum, associated with impaired spatial memory and learning. S1P possesses strong neuroprotective properties that are essential for maintaining normal neuronal excitability and synaptic transmission in hippocampal cells. Within the brain, S1P controls apoptosis, neuronal growth, synaptic plasticity, and glial cell activation (71). S1P signaling plays a role in synaptic dysfunction associated with T2DM, highlighting the considerable influence of this pathway on both neuronal health and diabetic complications.

Studies in animals have shown that increased brain S1P levels can alleviate spatial memory deficits in STZ-induced diabetic rats, potentially by inhibiting ceramide depletion in the hippocampus (72). Notably, in the initial stages of Alzheimer’s disease, S1P levels decrease in the hippocampus and prefrontal cortex (73). Moreover, experimental research suggests that the development and progression of diabetes-related cognitive dysfunction are tightly associated with S1PR2 (72). S1PR2, the principal receptor for S1P and a major regulator of neuroinflammation, has a critical role in learning and memory functions (74).

IP is used as a serum biomarker for mild cognitive impairment associated with diabetes in rodent models. In randomized controlled trials, S1P has been shown to effectively improve blood vessel function and significantly enhance neurovascular coupling (52), thereby alleviating cognitive dysfunction in diabetic patients. Clinical studies indicate that in type 2 diabetic patients with cardiovascular autonomic neuropathy, plasma S1P levels are significantly lower compared to those without CAN (75), with a negative correlation observed exclusively in female type 2 diabetic patients. Furthermore, the stromal cell-derived factor and the S1P agonist can affect diabetic wound healing and diabetic painful neuropathy. Diabetic painful neuropathy is a common complication of diabetes. MicroRNA-130a-3p targets S1PR1, leading to the activation of microglia and astrocytes, which in turn promotes apoptosis and oxidative stress in primary neurons exposed to high glucose levels (76). Experimental studies suggest that Sphk1 and TNF-α are involved in the development of peripheral neuropathic pain in streptozotocin-induced diabetic rats, underscoring the intricate interactions of biochemical factors in diabetic neuropathy and potential therapeutic targets (77).

## 5 Conclusion and future directions

In conclusion, S1P exhibits multifaceted and varied roles across various stages, forms, and complications of diabetes through the activation of the S1P/S1PR axis and interactions with signaling molecules like Akt and MAPK. The activation and interaction of these signaling pathways are essential for preserving islet cell function, modulating insulin sensitivity, maintaining metabolic balance, and controlling inflammation and the development of complications. A deeper comprehension of these complex signaling networks will enable the creation of innovative therapies targeting the S1P pathway, offering possibilities for treating and preventing diabetes and its associated complications.
