# Supplementary material for: Sphingosine-1-phosphate in the regulation of diabetes mellitus: a scientometric study to an in‐depth review
Source: Front Endocrinol (Lausanne). 2024 Dec 24;15:1377601. doi: 10.3389/fendo.2024.1377601 (PMC11703751; doi:10.3389/fendo.2024.1377601)
Supplement: Supplementary file 1 [file Table1.docx]

**Supplementary table 1:** abbreviation list

| Rank | abbreviation | full title | Rank | abbreviation | | full title |
| --- | --- | --- | --- | --- | --- | --- |
| 1 | AGEs | advanced glycosylation end products | 30 | | INS-1 | rat islet cell tumor |
| 2 | Akt | Protein kinase B | 31 | | IPC | ischemic preconditioning |
| 3 | ApoM | Apolipoprotein M | 32 | | IR | insulin resistance |
| 4 | AT | Adipose tissue | 33 | | IRS | insulin receptor substrate |
| 5 | ATM | adipose tissue macrophages | 34 | | MAPK | mitogen-activated protein kinase |
| 6 | BRB | blood-retinal barrier | 35 | | MKP-3 | Mitogen-activated protein kinase phosphatase 3 |
| 7 | cAMP | Cyclic adenosine monophosphate | 36 | | NF-κB | nuclear factor kappa-B |
| 8 | CAN | cardiovascular autonomic neuropathy | 37 | | NOD | Non-obese diabetic |
| 9 | Cers | Ceramide synthase | 38 | | PDR | proliferative diabetic retinopathy |
| 10 | CTGF | connective tissue growth factor | 39 | | PI3K | Phosphatidylinositol 3-kinase |
| 11 | DKD | diabetic kidney disease | 40 | | PKA | protein kinase A |
| 12 | DM | diabetes mellitus | 41 | | PP2A | Protein Phosphatase 2A |
| 13 | DMMCI | diabetic mild cognitive impairment | 42 | | PPARγ | peroxisome proliferator-activated receptor γ |
| 14 | DN | Diabetic nephropathy | 43 | | ROS | reactive oxygen |
| 15 | DPN | diabetic peripheral neuropathy | 44 | | S1P | Sphingosine-1-phosphate |
| 16 | DR | Diabetic Retinopathy | 45 | | S1PR | Sphingosine Kinase 1 receptor |
| 17 | ECs | endothelial cells | 46 | | SDF | stroma-derived factor |
| 18 | EMT | Epithelial-Mesenchymal Transition | 47 | | SGPL1 | sphingosine-1-phosphate lyase |
| 19 | ERK1/2 | Extracellular regulated protein kinase | 48 | | SphK1 | Sphingosine Kinase 1 |
| 20 | ESRD | end-stage renal disease | 49 | | SphK2 | Sphingosine Kinase 2 |
| 21 | FFA | Free fatty acids | 50 | | STZ | Streptozotocin |
| 22 | GLUT1 | glucose transporter type 1 | 51 | | T1DM | Type 1 Diabetes Mellitus |
| 23 | GLUT4 | glucose transporter type 4 | 52 | | T2DM | Type 2 diabetes Mellitus |
| 24 | GSK-3 | Glycogen synthase kinase-3 | 53 | | TER | transmonolayer resistance |
| 25 | HFD | high-fat diet | 54 | | TGF-β | transforming growth factor-β |
| 26 | hRVECs | human retinal vascular endothelial cells | 55 | | TNF-α | tumor necrosis factor-α |
| 27 | IL-1β | Interleukin-1β | 56 | | VEGF | vascular endothelial growth factor |
| 28 | IL-6 | Interleukin-6 | 57 | | WoSCC | Web of Science Core Collection |
| 29 | IL-8 | Interleukin-8 | 58 | | ZO-1 | zonula occludens-1 |
